# Ultrafast Laser Patterning of Metals Commonly Used in Medical Industry: Surface Roughness Control with Energy Gradient Pulse Sequences

**DOI:** 10.3390/mi14020251

**Published:** 2023-01-19

**Authors:** Luca Leggio, Yoan Di Maio, Alina Pascale-Hamri, Gregory Egaud, Stephanie Reynaud, Xxx Sedao, Cyril Mauclair

**Affiliations:** 1 Laboratoire Hubert Curien, Université Jean Monnet, 18 Rue Professeur Benoît Lauras, 42000 Saint-Étienne, France; 2 GIE Manutech-USD, 18 Rue Professeur Benoît Lauras, 42000 Saint-Étienne, France

**Keywords:** ultrafast lasers, roughness, medical applications, surface topography, material processing, surface ablation

## Abstract

Ultrafast laser ablation is widely used as a versatile method for accurate micro-machining of polymers, glasses and metals for a variety of industrial and biomedical applications. We report on the use of a novel process parameter, the modulation of the laser pulse energy during the multi-scan texturing of surfaces. We show that this new and straightforward control method allows us to attain higher and lower roughness ($R_{a}$) values than the conventional constant pulse energy irradiation sequence. This new multi-scanning laser ablation strategy was conducted on metals that are commonly used in the biomedical industry, such as stainless steel, titanium, brass and silver samples, using a linear (increasing or decreasing) gradient of pulse energy, i.e., varying the pulse energy across successive laser scans. The effects of ablation were studied in terms of roughness, developed interfacial area ratio, skewness and ablation efficiency of the processed surfaces. Significantly, the investigation has shown a global trend for all samples that the roughness is minimum when a decreasing energy pulse sequence is employed, i.e., the irradiation sequence ends up with the applied laser fluences close to threshold laser fluences and is maximum with increasing energy distribution. Scanning electron microscopy (SEM) and atomic force microscopy (AFM) analysis on single craters with the three different energy deposition conditions revealed a chaotic and random material redistribution in the cases of uniform and increasing energy distributions and the presence of regular laser-induced periodic surface structures (LIPSS) at the bottom of the ablation region in the case of decreasing energy distribution. It is also shown that the ablation efficiency of the ablated surfaces does not significantly change between the three cases. Therefore, this novel energy control strategy permits the control of the roughness of the processed surfaces without losing the ablation efficiency.

## 1. Introduction

Alteration of metal surfaces induced by laser irradiation was very soon observed after the invention of the laser [1]. The morphology of the textured surfaces has been proved to be directly correlated to the fluence applied and to the accumulation effects [2]. Femtosecond laser ablation is a precise micro-machining technique that was tested for the first time in the mid-1990′s to ablate micrometric features on silica and silver surfaces with low thermal damage [3,4]. Afterward, it has been extensively investigated for the ablation of metallic surfaces in a wide variety of applications, including surgery, biology, tribology, wettability, color marking and cutting [5,6,7,8,9,10,11,12,13,14,15].

Femtosecond lasers have also been employed to control the surface roughness with the generation of laser-induced micro- and nano-structures, such as microbumps and nanojets [16], microspikes and microgrooves [17], LIPSS (Laser-Induced Periodic Surface Structures) [18,19], holes and micropillars [20]. These structures of a wide variety and multiple scales are achieved by finely tuning several laser parameters, such as the pulse number, energy, polarization and repetition rate for a given material.

Likewise, researchers have investigated more advanced laser-tuning parameters, such as the laser spot spatial energy distribution with, e.g., top-hat beams [21]. Moreover, the effect of the temporal energy deposition rate has been thoroughly studied. Detrimental heat accumulation effects at MHz repetition rates with the onset of heat-affected zones (HAZ) surrounding the ablated area have been reported [22,23,24]. Further developments using short bursts of pulses at the GHz level [25,26,27] have shown an improved ablation efficiency with restricted heat-affected zones [28,29,30]. There, the number of pulses within the burst directly affects the final ablated zone’s roughness [31,32,33]. Researchers have recently shown the importance of the surface scanning scheme (overlap, the crossover of parallel scans, etc.) to master the final surface rugosity further [34,35].

The control of the roughness has direct consequences on the local wettability, with the possibility of achieving hydrophobic or hydrophilic surfaces [36,37,38,39]. This possibility of tuning wettability is of utmost importance for various tribological, nautical and biomedical applications. In the latter one, the fabrication of surfaces with added functions has many applications, such as cell adhesion, differentiation [5], anti-bacterial [40,41], self-cleaning, anti-fouling [42] and icephobic properties [43]. All these functions are seamlessly related to the control of the processed surface roughness.

In this report, we present a novel and straightforward method to control the final surface roughness by altering the pulse energy during multi-scanning irradiation, which has not yet been discussed in the literature to the best of our knowledge. Unlike conventional laser processing, where a constant fluence was employed during the process, here we showcase that controlling the energy level within the multi-pulse irradiation sequence permits the control of the surface’s final roughness with a nearly unchanged ablation efficiency.

Although the variation of the pulse energy was manually carried out between successive scan passes in this proof-of-concept report, the control of the pulse energy during laser processing can be easily automated by tuning the laser’s built-in external acousto-optic modulator; hence, a direct adaptation to industrial processing applications is straightforward.

A classic high-overlap raster scan pattern of 20 scans using uniform laser pulse energy was compared to the same scan pattern, with the pulse energy varying linearly between the scans with either an increasing or decreasing trend and the overall energy deposition being identical. The surface roughness after irradiation was subsequently evaluated for each case. This parametric study was conducted on four materials: stainless steel, titanium, brass and silver. The surface topography of the irradiated surfaces was analyzed using confocal microscopy. Single-static shots at the three different conditions were equally achieved and characterized with atomic force microscopy (AFM) and scanning electron microscopy (SEM).

The presence of regular LIPSS at the bottom of the ablation region was noticed in the case of decreasing energy configuration. Topographic parameters as the roughness $(R_{a}$), the developed interfacial area ratio $(S_{dr}$), the skewness $(R_{sk}$) and the ablation efficiency of the irradiated surfaces was measured. It was observed that changing the energy distribution during the irradiation permits the increase/reduction of the final roughness up to +17%/−25% compared with the usual constant energy sequence for the case of titanium, with similar trends on the other materials investigated here. This result can be significant for biomedical applications where control of the surface roughness is required.

This paper is structured in the following way: Section 2 provides a detailed description of the femtosecond laser setup, the scanning methodology employed in the experiments, as well as of the metal workpieces. Section 3 briefly introduces the topographic parameters ($R_{a}$, $S_{dr}$, $R_{sk}$) and presents their values on the processed scanned surfaces showing the roughness control with the pulse energy gradient. Ablated zones from static irradiations with identical energy distributions are also investigated under AFM and SEM, providing further insights into the laser–matter interaction specificities in the gradient case. Section 4 provides some concluding remarks.

## 2. Experimental Details

**Figure 1:**
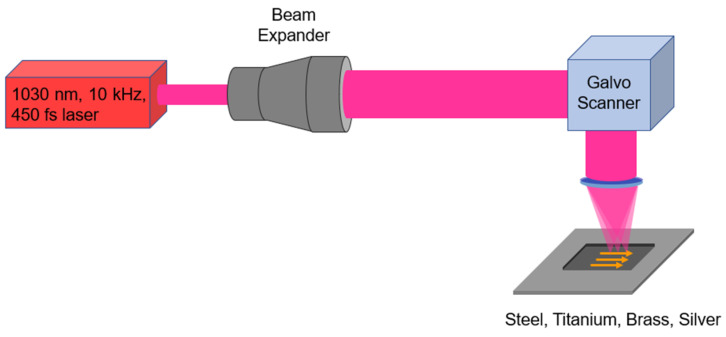
Schematic of the experimental setup: the beam is expanded and then focused on the surface of a metallic workpiece by a galvo scanner equipped with a 100 mm *f*-theta lens. A linear polarization parallel to the scanning direction (orange arrows) has been used. The inset shows the usual raster scanning pattern with a spot diameter of 2ω = 26 µm and a spacing of $\Lambda_{x}$ = $\Lambda_{y}$ = 3.64 µm, yielding a spatial pulse overlap OL = 86% in both x and y directions.

The schematic diagram of the experimental setup for laser ablation of metallic workpieces is shown in Figure 1. The micro-machining was carried out using a femtosecond laser system (Tangor model from Amplitude Laser Group, Pessac, France) with a central wavelength of 1030 nm, a repetition rate fixed at 10 kHz, an adjustable average power with a maximum value of 100 W and a pulse duration of 450 fs at full–width half maximum.

The linearly polarized gaussian beam from the fs laser was magnified through a beam expander (BE) and then sent through a galvo scanner (ProSeries II Scan Head from Cambridge Technology, General Scanning Solutions, Phoenix, AZ, USA) and focused on the surface of a metallic workpiece through a 100 mm *f*-theta objective.

The focused laser beam diameter at the focal point has been adjusted by adapting the magnification power of the beam expander to a value of 2ω = 26 μm (at 1/e^2^), measured using a Beamage-4M camera (from Gentec-EO, Québec, QC, Canada). For all micro-machined surface areas, a constant number of pulses per unit area were deposited on the metal surface using the common raster scanning method (see Figure 1).

Rectangles with defined size (4 × 2 mm [44] for stainless steel, brass and silver, or 2 × 1 mm rectangle in the case of titanium) have been achieved with pulse overlap OL of 86% in both dimensions, following the optimum irradiation conditions for reduced roughness described in [44]. This corresponds to a spacing of $\Lambda_{x}=\Lambda_{y}=2\omega \left(1-OL\right)=3.64\;µm$, considering a scan speed of 36.4 mm/s and a pulse repetition rate of 10 kHz. The materials were 1 mm thick samples of 316L stainless steel, titanium alloy TA6V, Cu-Zn alloy (commonly known as brass) and silver, which are generally used in biomedical and medical industry applications. The samples have been fixed on a three-axis linear motion stage (model MLJ150/M from Thorlabs Inc., Newton, NJ, USA) and placed at the focal plane of the galvo scanner *f*-theta lens.

**Figure 2:**
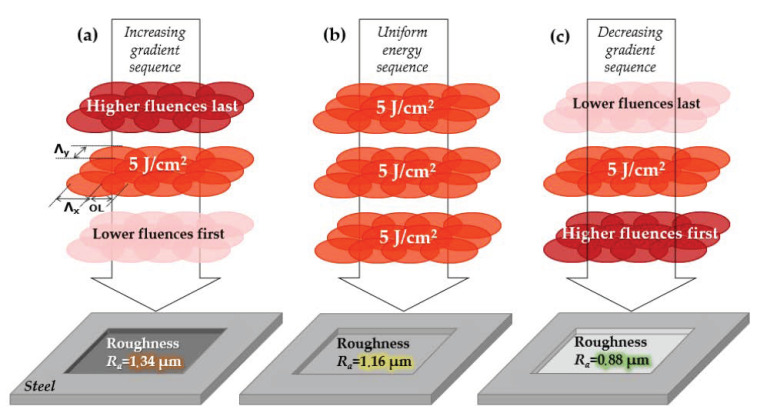
Schematic representation of the irradiation sequences consisting of 20 repetitions of the same scan pattern (only three are drawn for clarity) with (**a**) an increase in laser fluence between the scans starting with low fluences (0.5 J/cm^2^) and ending with high fluences (9.5 J/cm^2^), (**b**) constant fluence at 5 J/cm^2^ and (**c**) a decrease in laser fluence starting with high fluences and ending with low fluences. The post-irradiation surface roughness for steel is indicated in each case showing a decrease of roughness from (**a**) to (**b**) and to (**c**).

On each sample, three rectangular areas with different laser irradiation sequences and an identical amount of total deposited energy have been conducted. The laser pulse fluence of 5 J/cm^2^ was chosen based on a previous study [44], where it was shown to be a good compromise between easy implementation, high throughput and relatively low surface roughness after the laser process. This value was also chosen in this way for the gradient study. It is believed that the fluence can be shifted to a higher or lower value if needed; therefore, an adapted gradient step would be required, and a similar observation would be again thereupon achieved. These energy irradiation sequences are schematically depicted in Figure 2. The increasing energy sequence (see Figure 2a) consisted of 10 steps of two scans where the laser fluence was increased between each two-scan step following this order: 0.5, 1.5, 2.5, 3.5, 4.5, 5.5, 6.5, 7.5, 8.5 and 9.5 J/cm^2^, i.e., the low fluences being applied first and the high fluences at the end. The uniform energy sequence was 20 scans with a constant peak fluence of 5 J/cm^2^ (see Figure 2b). Finally, the decreasing energy sequence (see Figure 2c) also consisted of 10 steps of two scans where the laser fluence was decreased between each two-scan step following this order: 9.5, 8.5, 7.5, 6.5, 5.5, 4.5, 3.5, 2.5, 1.5 and 0.5 J/cm^2^, i.e., the high fluences being applied first and the low fluences at the end. We underline here that the same total amount of laser fluence was kept in all three cases.

**Figure 3:**
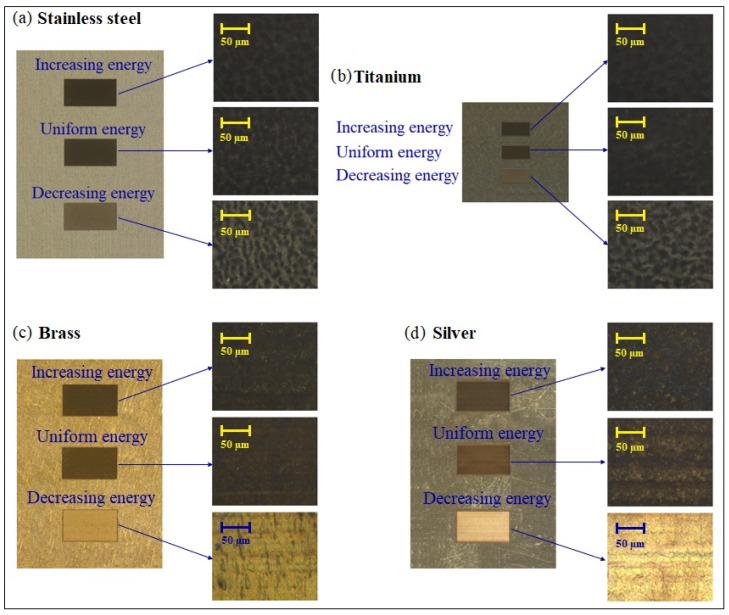
Shows 10× and 100× optical microscopic images of the workpieces. The rectangle areas have been irradiated on an area of 4 × 2 mm for steel, brass and silver and 2 × 1 mm for titanium. Interestingly, the brightest areas, corresponding to the decreasing gradients, also exhibit the lowest values of roughness.

**Figure 4:**
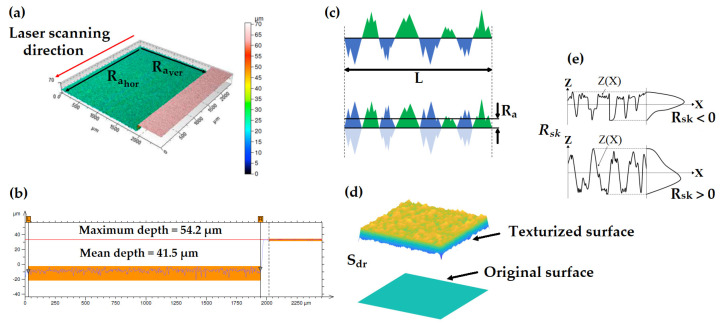
Example of measurement with profilometer applied to stainless steel in the case of increasing gradient: (**a**) reconstructed 3-D surface profile of the textured surface where the laser scanning direction is specified. The overall roughness is averaged between the horizontal roughness $\;R_{a\;hor\;}$(parallel to the scanning direction) and the vertical roughness $\;R_{a\;ver\;}$ (perpendicular to the scanning direction) and over three measurement repetitions, (**b**) measurement of the depth after ablation, (**c**) schematic illustration of the definition of roughness$\;R_{a}$, (**d**) developed interfacial area ratio $S_{dr}$ and (**e**) skewness of the roughness profile $R_{sk}$. See text for details.

**Figure 5:**
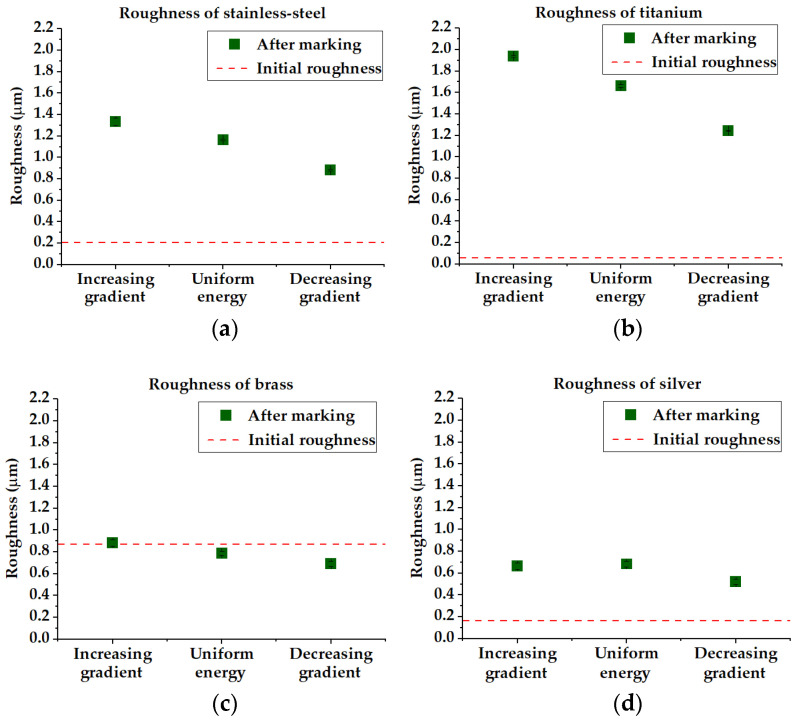
Roughness $R_{a}$ of (**a**) stainless steel, (**b**) titanium, (**c**) brass and (**d**) silver. The roughness data after laser patterning are plotted with the initial roughness. Steel, titanium and silver exhibit an increase in roughness post-irradiation, whereas brass keeps nearly the same level of roughness. In the cases of steel, titanium and brass, the highest value of roughness is obtained with an ascending energy gradient (fluences from 0.5 to 9.5 J/cm^2^) and reaches the lowest value with a descending energy gradient (fluences from 9.5 to 0.5 J/cm^2^). In the case of silver, the roughness with an increasing energy gradient is slightly lower than in the case of uniform energy.

**Figure 6:**
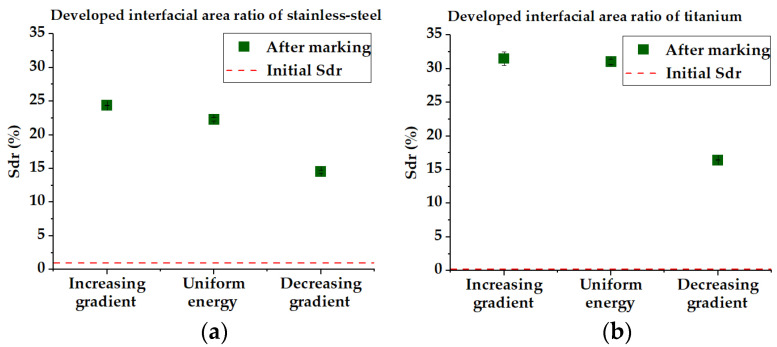

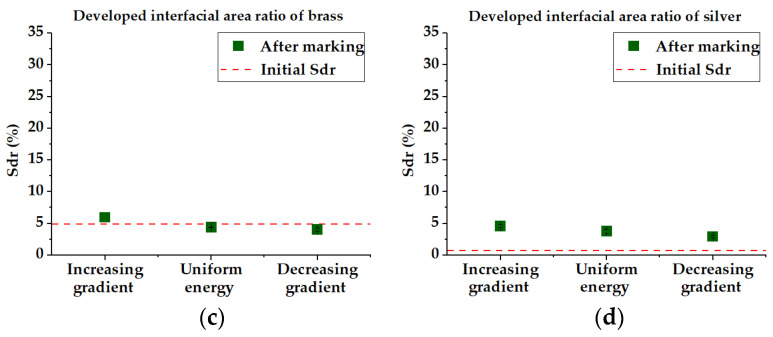
$S_{dr}$ parameters of (**a**) stainless steel, (**b**) titanium, (**c**) brass and (**d**) silver. The trend is quite similar to the roughness parameter $R_{a}$.

**Figure 7:**
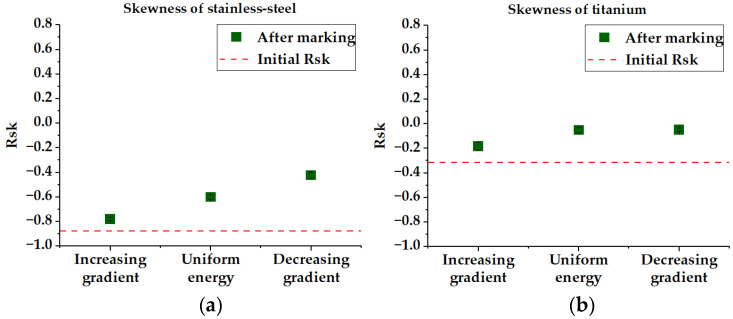
$R_{sk}\;$parameters of (**a**) stainless steel and (**b**) titanium. The $R_{sk}=0$ corresponds to a symmetrical profile.

An ultrasonic bath with acetone and then ethanol at room temperature was applied to clean the surfaces of the workpieces after engraving. The workpieces have been elaborated and characterized three times for each case to assess their repeatability. They have been observed under optical microscopy with 10× and 100× magnifications (Figure 3) using the same exposure configuration. This optical microscope inspection permits us to already note that the color of the surfaces is much shinier in the cases of decreasing energy, especially in the cases of brass and silver. As discussed in the next section, this can be linked to lower surface roughness. In a subsequent step, the specimens were analyzed for roughness and depth with a confocal microscope (model Altisurf 530 from Altimet, Marin, France) equipped with a probe (model Chrocodile S from Precitec, Gaggenau, Germany). Its lateral and axial spatial resolutions are 1.8 µm and 30 nm, respectively, with a scanning step of 2 µm and a 1 mm measurement range. The ISO 4288 procedure for roughness measurements was applied with the same Gaussian cutoff filter for all the samples. An example of surface topography is shown in Figure 4a,b for a sample of stainless steel in the case of increasing energy distribution. The main roughness measurement data presented on the curves in this paper (Figure 5, Figure 6 and Figure 7) are available as numeral values in the supplementary section in Appendix A.2.

## 3. Results and Discussion

### 3.1. Topographic Parameters: Roughness, Developed Interfacial Area Ratio and Skewness

The topographic surface characterization of the processed sample is one of the post-processing steps that deserve consideration [45,46,47]. The first parameter to be analyzed is the arithmetical mean deviation of the measured profile, which is better known as the roughness parameter $R_{a}$ (expressed in µm, eq. in ref. [48]). The value of $R_{a}\;$indicates the average surface roughness measured over the length of the sample, which means the average difference between peaks and valleys. The areas of the graph below the central line (i.e., the valleys) are projected above the central line, which determines the level of the initial surface (see Figure 4c). The value of $R_{a}\;$is the mean height of the resulting profile; in other terms, it is the average roughness. The greater the difference between the peaks and valleys, the higher the roughness of the surface is. Other topographic parameters that deserve investigation are the developed interfacial area ratio $S_{dr}\;$and the skewness of the roughness profile$\;R_{sk}$, whose mathematical definitions are reported in the literature [48].

Literally, the $S_{dr}\;$is defined as the complexity of the surface in terms of the percentage of the textured surface area compared to the corresponding planar area (see Figure 4d) [48]. In other words, it is the percentage of the surface affected by peaks and valleys. The complexity of a completely flat surface is expressed as $S_{dr}=0\%$. When a surface has any slope, its $S_{dr}$ value becomes comparatively larger ($S_{dr}>0\%,$ see Figure 4d). The skewness of the roughness profile $\;R_{sk}\;$indicates the mean deviation of a surface profile (i.e., the height distribution) compared with the mean line that determines the initial flat surface:$\;R_{sk}=0$ is symmetric over the mean line, $\;R_{sk}>0$ indicates a mean deviation below the mean line and $\;R_{sk}<0$ indicates a mean deviation above the mean line (see Figure 4e). In other words, $R_{sk}\;$is the asymmetry factor of the profile. It gives information on the morphology of the surface state: $R_{sk}>0$ describes a peaked surface altogether, $R_{sk}<0$ suggests a near-plateau surface, and when $R_{sk}\ll 0$, it means that the surface has deep pores. A surface with a sinusoidal profile has symmetric topography and $R_{sk}=0$ [48].

These parameters have been extracted from surface topography measurements performed with the aforementioned confocal microscope. The measurements of the roughness parameter $R_{a}$ (µm) for the abovementioned materials are plotted in Figure 5 for the cases of uniform energy and increasing/decreasing energy distributions and are averaged between horizontal and vertical roughness over three measurement repetitions. Linear polarization parallel to the scanning direction has been used for the creation of rectangles. As we can appreciate from Figure 5, an increase in post-irradiation roughness ($R_{a})$ is observed compared with the initial surface roughness (that was also measured with the same apparatus) in the cases of stainless steel, titanium, silver and also brass to a lower extent because of a relatively high initial roughness.

The roughness of the stainless steel and titanium samples exhibits a clear trend, with the maximum value noticed with the increasing energy distribution (1.336 µm and 1.941 µm, respectively) and the minimum one with the case of decreasing energy (0.881 µm and 1.244 µm, respectively). In the case of brass, whose initial roughness is significantly higher than the other samples, the trend is found to be similar to the previous cases, but the values vary in a much smaller range (0.884–0.691 µm). In the case of silver, the trend is somewhat different. The roughness with increasing energy gradient is slightly lower than in the case of uniform energy (0.666 µm and 0.684 µm, respectively) and a slight drop to 0.521 µm is observed in the case of decreasing energy. Thus, these results demonstrate that the use of the energy gradients permits better control of the final roughness. Noteworthy, the case of brass shows a situation where the femtosecond laser irradiation with decreasing energy distribution even permits the reduction of the surface roughness as compared with its initial value. Figure 6 focuses on the developed interfacial area ratio ($S_{dr})$. This parameter presents a nearly identical tendency as for the $R_{a}$ of all the workpieces. This means that the laser treatment implies an augmentation of the interfacial surface that is also greater in the case of increasing energy distribution. The decreasing energy gradient also involves a decrease of the $S_{dr}$. This parameter is of interest for applications where the interfacial area plays a significant role, e.g., in heat transfer, cell adhesion or anti-bacterial properties. We again note the particular case of brass where the laser treatment permits the reduction of the $S_{dr}$ from its relatively high initial value.

Concerning the skewness $R_{sk}$, some results are depicted in Figure 7. If no global trend could be found among all the materials, the results on steel and titanium seem to show that the decreasing gradient or uniform energy permit rendering the surface more symmetrical ($R_{sk}$ trends to 0, i.e., close to a sinusoidal profile).

In the cases of brass and silver, no detectable trend has been found concerning the $R_{sk}$. Having in mind that a negative $R_{sk}$ value implies a spikier surface with isolated peaks (see Figure 4e), this quick observation is of interest for applications where this type of corrugation could be optimized for a greater impact on anti-bacterial or superhydrophobic functions [40].

### 3.2. Ablation Efficiency

**Figure 8:**
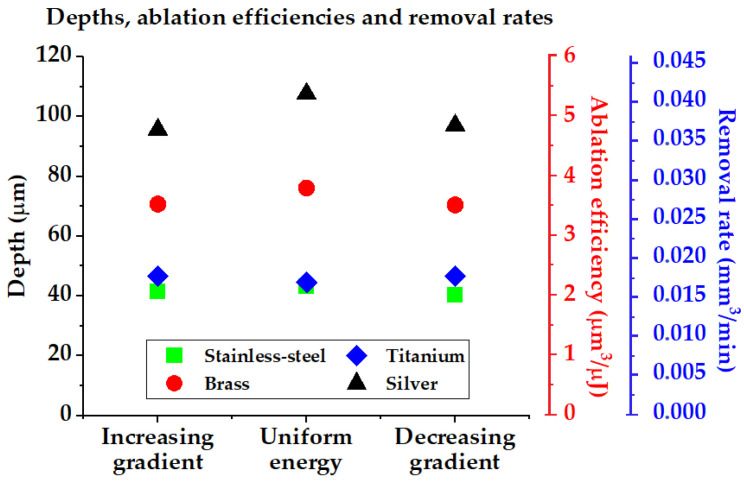
Experimental depths, ablation efficiencies and removal rates of the ablated surfaces on stainless steel, titanium, brass and silver. The use of ascending or descending pulse energy sequence does not significantly affect the ablation performance.

By measuring the depth of the ablated surfaces (averaged between three repeated measurements), the removal rate *RR* in mm^3^/min can be calculated following RR=V/t, with V being the ablated volume in mm^3^ and t the processing time in minutes. Likewise, the ablation efficiency *AE* in µm^3^/µJ can be deduced from $AE=V/\left(\;E_{p}N_{tot}\right)$, with V the ablated volume in µm^3^, $E_{p}$ the mean pulse energy in µJ and $N_{tot}$ the total number of laser pulses. As shown in Figure 8, it can be observed that the ablation efficiency and removal rate remain quasi-unaltered by the use of ascending, descending or uniform energy sequences during the ablation for all the material investigated here.

This shows that it is possible to control the surface roughness without compromising the ablation efficiency. Further validation of this result has been studied from ablation rate curves of the investigated materials found in the literature (Table 1). The experimental ablation rate curves obtained from static irradiation found in [49,50,51,52] were fitted with x-power functions (or logarithmic functions) in order to obtain an empirical relationship of the ablation depth per pulse with respect to the laser peak fluence.

The choice of the fitting equation was carried out to obtain the best fitting performance. The lasers used in these studies were relatively similar to the one employed here, lying in the infrared region with sub-picosecond pulse durations. In order to compare these static shot experimental data with our results, we have evaluated the number of pulses per area spot to be $N_{spot}\sim \;320$, taking into account the pulse overlap using our experimental conditions with the coefficient derived for gaussian distribution as in [53] and the total number of scan repetitions (20); some details are provided in the supplementary section (Appendix A.1). Thus, a rapid estimation of the total ablated depth for a uniform energy with a constant peak fluence of 5 J/cm^2^ is obtained for a material by simply multiplying $N_{spot}$ by the results of the equations in Table 1. For the case of energy gradient, as 10 different peak fluences are employed, the same estimation has been conducted for each of the 10 peak fluence values with a multiplication of $N_{spot}$/10.

As can be seen in Table 1, the depth results calculated from the literature are in relatively good agreement with our measurements, with a slight underestimation, as mentioned in Appendix A.1. We point out here that this comparison has to be taken with care as one cannot directly compare ablation depth obtained through laser milling with depths obtained by static irradiations.

However, in a first approximation, these results appear to be coherent. Moreover, simple static ablation models are often used to discuss milling experiments in good agreement [54]. Overall, the ablation depths remain nearly constant despite the use of uniform and/or gradient energy irradiation sequences. As expected, the depth estimations based on the literature yield slightly lower ablation depths in the case of gradients when compared with the uniform sequence on all materials, which is also verified in our experiment, with the exception of Ti. Indeed, varying the peak fluence from 0.5 to 9.5 J/cm^2^ implies ablation regimes of low-removal efficiencies, e.g., when the laser fluence is very close to the threshold or very far from the optimal ablation fluence. Thus, it is slightly less efficient than a constant energy irradiation at a peak fluence which is close to the optimum ablation value.

### 3.3. Micrometric Analysis

Lastly, we tried to isolate the effects of the gradients from those of the scanning on metallic surfaces by investigating the static impacts at a micrometric scale by sending 20 laser pulses with and without the energy gradient sequence. The low number of 20 pulses was chosen in order to obtain relatively superficial impacts to ease the micrometric characterization.

**Figure 9:**
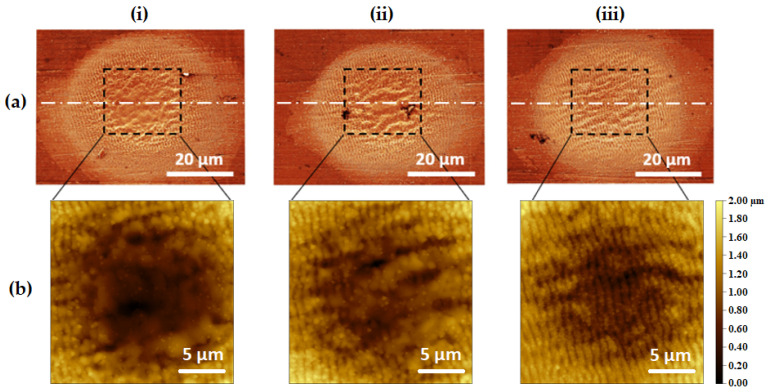
(**a**) SEM pictures (false color) of the static shots performed on stainless steel with a scale bar of 20 µm, (**b**) AFM characterization of the 20 × 20 µm central zone for each impact of (**a**) with a scale bar of 5 µm: (**i**) increasing gradient with 20 pulses distributed in the range 0.5–9.5 J/cm^2^, (**ii**) uniform energy with 20 pulses at 5 J/cm^2^, (**iii**) decreasing gradient with 20 pulses distributed in the range 9.5–0.5 J/cm^2^.

Thus, an analysis of the static shots on a stainless steel sample has been performed with SEM and AFM, as depicted in Figure 9a,b, respectively. The microscopic pictures of the shots are reported in the cases of uniform energy and increasing/decreasing energy distributions. From the SEM pictures, it can be seen that the overall spatial extent of the laser impacts is similar. We have equally observed that the mean ablation efficiency is also similar in all cases. The closer topographic observation offered by the AFM analysis in Figure 9b permits the revealed differences in the interaction results. In the case of the uniform sequence (Figure 9b(ii)), we observe a stochastic reorganization of the surface at the micrometric scale, which is typical of the strong ablation regime on steel where the energy coupling is governed by electronic heat diffusion (>5 J/cm^2^), as observed in [49].

In the case of increasing energy distribution (Figure 9b(i)), this effect is even more pronounced as the latest pulses approach the maximum fluence level of 9.5 J/cm^2^ of this study. Conversely, in the case of decreasing energy distribution (Figure 9b(iii)), the laser ablation begins in the strong ablation regime and ends up in the gentle regime [49], approaching the close-to-threshold value of 0.5 J/cm^2^.

For this reason, a regular pattern of LIPSS has been formed in this case [49]. This result contrasts with the general observation that the LIPSS pattern tends to vanish under a large number of pulses [55,56]. However, this LIPSS vanishing effect occurs for pulses of constant energy, contrary to the decreasing energy sequence that ends with low-fluence irradiation, thus yielding the associated LIPSS pattern. Indeed, surface structuring, including a last low-fluence scan to add a sub-micrometric corrugation, can be advantageously used for super-hydrophobicity functions [57].

In the decreasing energy sequence, hypothetically, the ablation sparsely occurs on the irradiation region, thus smoothing out the rough surface left by the previous pulses of higher energy. While scanning (such as in Section 3.1), this smoothening effect observed in a static shot can be achieved on large surface areas, as the scanning experiments have shown, with lower post-irradiation roughness in the case of the decreasing energy (see Figure 5 and Figure 6). The chaotic local reorganization observed in the cases of uniform and increasing energy distributions is also coherent with the higher roughness obtained in the scanning experiments (see Figure 5 and Figure 6). Noteworthy, the higher roughness is achieved with the increasing energy irradiation sequence for static shots as well as for scanning processing.

## 4. Conclusions

A new approach for controlling the post-irradiation roughness of several metal workpieces by changing the laser energy during multi-scan irradiation has been presented. To the best of our knowledge, this method has not been discussed before in the previous literature, where instead, common approaches with uniform energy have been used. Pulse overlaps of 86% and a scan speed of 36.4 mm/s have been used for the experiments at 10 kHz of pulse repetition rate. The experiments showed the same trend for all the cases: the highest value of roughness has been obtained with an increasing pulse energy distribution, and the lowest one with the case of decreasing energy. Therefore, in some cases, it is more convenient to use energy gradients rather than the usual uniform energy irradiation, depending on the need to achieve higher or lower roughness. Likewise, an increasing energy distribution is evidently indicated for achieving a higher roughness compared with the case of uniform energy and a decreasing one for a lower roughness formation. Additional topographic parameters, such as the developed interfacial area ratio, the skewness and the ablation efficiency of the processed surfaces, have been investigated as well in this work. It has also been demonstrated that the ablation efficiency does not significantly change between uniform energy and energy gradients, which means that the ablation efficiency has not deteriorated from one case to another. Lastly, a static analysis has been performed with static shots to analyze the impact of the energy distributions on the topography of the steel surface at a micrometric scale by using an SEM/AFM tool. The irradiated steel surface exhibits a chaotic topography in the cases of uniform and increasing energy distributions of energy, whereas a smoother surface with LIPSS presence is observed in the decreasing energy case, in harmony with the aforementioned roughness trend observed in the scanning study. The outcomes shown in this work open new possibilities for roughness control in industrial and biomedical applications where tuning the surface topography is of utmost importance for achieving desired surface properties, such as hydrophobicity, self-cleaning, anti-bacterial effects and so on.

## Figures and Tables

**Table 1:** Comparison of the experimentally measured depths with calculated depths from ablation rate-fitting curves for the materials involved in this study based on the literature: *d* is the single pulse ablated depth (in nm) and *F* is the peak fluence in J/cm^2^. The estimated depth in µm was calculated for 320 pulses from the fitting equations in the case of uniform and gradient energy irradiations and compared with the experimental depth measurements.

Material	Fitting Curve	Estimated Depth (µm)		Measured Depth (µm)		
		Uniform	Increasing/Decreasing	Increasing	Uniform	Decreasing
Stainless Steel [49]	$d=40.783*F^{0.6372}$	36.9	35.8	41.6	43.2	40.3
Titanium [50]	d=18.57∗ln(F)+82.86	36.6	34.9	46.7	44.5	46.7
Brass [51]	d=32.32∗ln(F)+143.76	63.4	60.6	70.8	76.1	70.5
Silver [52]	$d=58.321*F^{0.9493}$	87.1	86.2	95.7	107.7	97.1

## Data Availability

The data that support the findings of this study are available from the corresponding author upon reasonable request.

## Appendix A

### Appendix A.1. Calculation of $N_{tot}$

Considering a scanning step of $\Lambda_{x}$=$\Lambda_{y}$= 3.64 µm in x and y for a beam diameter of 2ω = 26 µm at 1/e^2^, the number of pulses seen per unit area is given by $N_{spot}=N_{scans}\pi \omega^{2}/\left(\Lambda_{x}\Lambda_{y}\right)$~800 [53]. This calculation would be correct if the laser spot had a uniform top-hat profile [21]. However, the laser spot profile follows a Gaussian distribution. This means that each time the surface point sees a laser pulse, it does not see its peak fluence but a fraction of it; thus, the total amount of deposited energy is overestimated. A correction factor can be applied to $N_{spot}$ to take this into account. As reported by Borowiec and Haugen [58], the correction factor *k* is 0.63 for a groove, as derived from the integration of the summed Gaussian profiles along the scanning line. In our case, considering a rectangular surface instead of a groove, this factor becomes $k=0.63^{2}\sim 0.4$. So, the total number of pulses seen by a point becomes $N_{spot}\sim 0.4\times 800$ ~ 320. We point out here that this calculation is an approximation that may underestimate the ablation depth in the case of milling processes, as discussed in [59].

### Appendix A.2. Roughness, Depth and Ablation Efficiency Measurements Data

Table A1, Table A2, Table A3, Table A4, Table A5, Table A6, Table A7, Table A8, Table A9, Table A10, Table A11 and Table A12 gather the roughness parameters $R_{a}$, $S_{dr}$ and $\;R_{sk}$, the ablation efficiencies are shown in the curves of the paper for all the materials of this study and the depths.

**Table A1:** Roughness and depth of steel.

Energy Distribution	Roughness (µm)	Depth (µm)
Increasing	1.336	41.560
Uniform	1.165	43.224
Decreasing	0.881	40.273
Initial $R_{a}$	0.205	-

**Table A2:** Roughness and depth of titanium.

Energy Distribution	Roughness (µm)	Depth (µm)
Increasing	1.941	46.741
Uniform	1.663	44.533
Decreasing	1.244	46.661
Initial $R_{a}$	0.055	-

**Table A3:** Roughness and depth of brass.

Energy Distribution	Roughness (µm)	Depth (µm)
Increasing	0.884	70.762
Uniform	0.788	76.124
Decreasing	0.691	70.470
Initial $R_{a}$	0.870	-

**Table A4:** Roughness and depth of silver.

Energy Distribution	Roughness (µm)	Depth (µm)
Increasing	0.666	95.666
Uniform	0.684	107.668
Decreasing	0.521	97.121
Initial $R_{a}$	0.166	-

**Table A5:** Ablation efficiencies and removal rates of steel.

Energy Distribution	Ablation Efficiency (µm^3^/µJ)	Removal Rate (mm^3^/min)
Increasing	2.08	0.0158
Uniform	2.16	0.0165
Decreasing	2.01	0.0154

**Table A6:** Ablation efficiencies and removal rates of titanium.

Energy Distribution	Ablation Efficiency (µm^3^/µJ)	Removal Rate (mm^3^/min)
Increasing	2.33	0.0178
Uniform	2.22	0.017
Decreasing	2.33	0.0178

**Table A7:** Ablation efficiencies and removal rates of brass.

Energy Distribution	Ablation Efficiency (µm^3^/µJ)	Removal Rate (mm^3^/min)
Increasing	3.53	0.027
Uniform	3.80	0.029
Decreasing	3.52	0.0269

**Table A8:** Ablation efficiencies and removal rates of silver.

Energy Distribution	Ablation Efficiency (µm^3^/µJ)	Removal Rate (mm^3^/min)
Increasing	4.78	0.0365
Uniform	5.38	0.041
Decreasing	4.85	0.037

**Table A9:** $S_{dr}$ and $R_{sk}$ of steel.

Energy Distribution	$S_{dr}\;(\%)$	$R_{sk}$
Increasing	24.367	−0.779
Uniform	22.297	−0.601
Decreasing	14.541	−0.424
Initial value	0.964	− 0.880

**Table A10:** $S_{dr}\;$ and $R_{sk}$ of titanium.

Energy Distribution	$S_{dr}\;(\%)$	$R_{sk}$
Increasing	31.496	−0.182
Uniform	31.043	−0.051
Decreasing	16.403	−0.049
Initial Value	0.170	−0.313

**Table A11:** $S_{dr}$ and $R_{sk}$ of brass.

Energy Distribution	$S_{dr}\;(\%)$	$R_{sk}$
Increasing	5.931	−0.224
Uniform	4.390	0.037
Decreasing	4.014	−0.090
Initial Value	4.889	−0.410

**Table A12:** $S_{dr}\;$ and $R_{sk}$ of silver.

Energy Distribution	$S_{dr}\;(\%)$	$R_{sk}$
Increasing	4.536	0.440
Uniform	3.771	0.122
Decreasing	2.909	0.079
Initial Value	0.679	−0.705
